# Competence and contributions of radiologists to cardiac CT and MR imaging across Europe

**DOI:** 10.1007/s00330-024-10741-4

**Published:** 2024-04-29

**Authors:** Marc Dewey

**Affiliations:** 1 Department of Radiology, Charité – Universitätsmedizin Berlin, corporate member of Freie Universität Berlin and Humboldt-Universität zu Berlin, Berlin, Germany; 2 Berlin Institute of Health at Charité – Universitätsmedizin Berlin, Berlin, Germany; 3 Berlin University Alliance, Berlin, Germany; 4 DZHK (German Centre for Cardiovascular Research) partner site Berlin, Berlin, Germany; 5 Deutsches Herzzentrum der Charité (DHZC), Berlin, Germany

Cardiovascular diseases remain the leading cause of mortality worldwide [1], emphasizing the crucial role of cardiovascular imaging modalities such as cardiac computed tomography (CT) and magnetic resonance (MR) imaging [2, 3]. Radiologists play a pivotal role in the accurate interpretation of these imaging studies, thereby influencing patient management and outcomes [4–13]. A large-scale analysis from the European Society of Cardiovascular Radiology (ESCR) registry addressed the status of radiologist competence in cardiac imaging across Europe and emphasized the need for standardization and collaboration to ensure optimal patient care [14].

## Main findings of the analysis of the ESCR registry analysis

### Significant increase in cardiac CT and MR examinations

Between 2011 and 2022, there was a notable surge in both cardiac CT and MR examinations across Europe, with a 4.5-fold increase in CT examinations and a 3.8-fold increase in MR examinations. This substantial rise underscores the growing importance of these imaging modalities in cardiovascular disease management and highlights the increasing demand for their utilization in clinical practice.

### Evolution of clinical indications

The data reveal the following main clinical indications for cardiac CT and MR imaging. Suspected coronary artery disease (CAD) emerged as the predominant indication (59%) for cardiac CT, followed by transcatheter aortic valve replacement planning (21%). Interestingly, there was a notable increase in the number of patients with intermediate pretest probability undergoing CT for suspected CAD from 61% in 2012 to 82% in 2022, indicating a further increase in appropriate clinical decision-making. For cardiac MR, suspected myocarditis (26%), CAD (21%), and suspected cardiomyopathy (19%) were the main indications, reflecting the diverse diagnostic capabilities of this imaging modality in assessing myocardial inflammation, ischemia, and structural abnormalities.

### Role of radiologists in reporting

The majority of CT and MR examinations were reported by radiologists (76% and 71%, respectively), underscoring their central role in the interpretation and diagnosis of cardiac imaging studies. However, a notable proportion of examinations were also reported in consensus with non-radiologists (19% and 27%, respectively), highlighting the importance of interdisciplinary collaboration in cardiac imaging. A small minority of CT and MR examinations (5% and 2%, respectively) were reported by non-radiological specialties or through separate readings of radiologists and non-radiologists.

**How do these findings fit into the current landscape?**

The field of cardiac imaging has witnessed rapid advancements in technology and techniques [15–17], enabling more accurate diagnosis and characterization of cardiovascular diseases. Cardiac CT and MR imaging have become indispensable tools in the evaluation of various cardiovascular and cardiothoracic conditions, including CAD, inflammation, myocardial ischemia, and cardiomyopathies. However, the interpretation of these imaging studies requires specialized knowledge and expertize, particularly in recognizing subtle anatomical and pathological findings and optimal use of novel technologies.

**What are the remaining key opportunities?**

An immense work has been done primarily by the European Society of Radiology (ESR) and the ESCR (in collaboration with national societies) with regard to further increasing the competence of radiologists in cardiac and cardiothoracic CT and MR imaging. Apart from the major congresses of the ESR and ESCR, these include initiatives to start education at the undergraduate level with the ESR ebook for undergraduate education with a dedicated chapter on Cardiac imaging [18], harmonizing radiology training through the European Training Curricula for general Radiology and subspecialisation training [19], the plethora of resources offered through the ESR Premium Education Package [20], the subspecialty diploma of the European Board of Cardiovascular Radiology (EBCR) [21], and availability of dedicated cardiovascular imaging fellowships through the European School of Radiology (ESOR) [22]. Great additional effort should be undertaken to further standardize protocols, implement artificial intelligence solutions, and promote consistent reporting [23, 24] through hands-on cardiac imaging courses.

Addressing these opportunities fully will benefit from multifaceted approaches involving the interdisciplinary collaboration of ESR and ESCR and other stakeholders, among them healthcare institutions, other professional societies, and regulatory bodies on the European and national levels. In addition, efforts should be made to establish additional clinical practice guidelines about cardiac MR [8] and CT [25] for image acquisition, interpretation, and reporting, thereby promoting high-quality consistency in clinical practice.

Furthermore, fostering collaboration among radiologists, general practitioners, cardiologists, and cardiac surgeons [10] is essential for enhancing interdisciplinary communication and facilitating optimal patient care. By continuing to establish multidisciplinary quantitative cardiovascular imaging meetings, further clinical and technical consensus publications will result [18, 19, 26] that will ultimately improve diagnostic performance, resource utilization, and patient outcomes.

**What about sharing data from this analysis?**

Importantly, the ESCR registry raw data underlying the original published article [14] is available upon request using office@escr.org for data sharing and exchange. It would be pivotal if the original source data underlying analyses such as the exciting previous analysis were more often shared with other researchers using data-sharing platforms such EUCAIM (www.eucaim.org) funded by the European Commission and/or the GUIDE-IT platform funded by German Research Foundation (DFG) (www.guide-it.org). This will ensure sustainable access to pivotal underlying information for the entire radiology community.

**What are the conclusions that can be drawn from this analysis?**

The competence and involvement of radiologists in cardiac CT and MR imaging play a critical role in the accurate diagnosis and management of cardiovascular diseases. Radiologists are central in the provision of cardiovascular imaging services and high-quality medical care in cardiovascular medicine. Suspected CAD and suspected myocarditis are the most common indications for cardiac CT and MR, respectively. The data from the ESCR registry also show a relevant increase by approximately a factor of four in cardiac CT and MR examinations in the last decade. Radiologists actively engaged in cardiovascular imaging play a central role in providing quantitative imaging findings as the basis for evidence-based recommendations for personalized optimal medical therapy and/or invasive treatment.

I would like to wholeheartedly congratulate the authors from the pivotal ESCR registry on providing important evidence using real-life data from Europe about the best use of cardiovascular imaging. We look very much forward to future analyses providing additional insights into the potential to further improve cardiovascular imaging and care.
